# Supplementary material for: Increased absorption in autonomous sensory meridian response
Source: PeerJ. 2020 Feb 17;8:e8588. doi: 10.7717/peerj.8588 (PMC7032055; doi:10.7717/peerj.8588)
Supplement: Supplemental Information 1 [file peerj-08-8588-s001.docx]

**Supplemental Results**

An additional analysis was conducted using the whole data set rather than just the subsample of ASMR-experiencers and control participants who were matched for age and gender, in order to establish whether similar results would be obtained. To this effect 10 participants whose age was over 3 SD away from the mean were deleted resulting in the total sample size of 306 (128 controls, 178 ASMR-experiencers). The groups differed significantly in terms of age t(303) = 6.350, p <.001 but not in terms of gender [χ*^(221, N = 306 )^*= 2.677, p = .262].

In order to establish whether there is a relationship between age and the constructs of interest Pearson’s Correlations were conducted which revealed a positive relationship between age and MAAS (r(305) = .157, p =.006) and between age and FES (r(125) = .238, p = .008) but a non-significant correlation was found between age and TAS (r (303) = .025, p = .663).

Therefore, an independent samples t-test was conducted on absorption (TAS) which revealed a statistically significant group difference t (304) = 4.691, p < .001, Cohen’s d = 0.55 with ASMR-experiencers scoring higher (M = 107.49, SD = 25.81) than controls (M= 94.23, SD = 22.26).

Subsequently, an ANCOVA with age included as a covariate was conducted on mindfulness (MAAS) which showed a non-significant effect of group F (1,302) = 0.020, p = .887, ŋp² = .000 suggesting no differences between ASMR-experiencers (M = 3.26, SD = 0.80) and controls (M = 3.32, SD= 0.85) with respect to this personality trait. Similar results were obtained by conducting ANCOVA on flow (FES) with age included as a covariate which also revealed a non-significant main effect of group (F (1, 122) = 0.036, p = .850, ŋp² = .000) with ASMR-experiencers scoring similarly (M = 38.64, SD = 8.99) to controls M = 37.90, SD = 8.19).

As such this additional analysis further confirms that the only statistically significant difference between ASMR-experiencers and controls is in terms of absorption on which ASMR-experiencers score higher than controls.
