# Supplementary material for: Increased absorption in autonomous sensory meridian response
Source: PeerJ. 2020 Feb 17;8:e8588. doi: 10.7717/peerj.8588 (PMC7032055; doi:10.7717/peerj.8588)
Supplement: Supplemental Information 2 [file peerj-08-8588-s002.docx]

**Appendix 1. Questionnaire**

**Demographic Questions**

What is your gender? (Female/Male/Other). If you selected Other, please specify:____

What is your age? ___

Do you attend Bath Spa University? Yes/No

**ASMR Questions**

Here is a description of a phenomenon called autonomous sensory meridian response (ASMR).

ASMR can be defined as a pleasurable tingling sensation which typically originates on the scalp and spreads down the spine and through the whole body which is typically induced by certain sounds (e.g. turning pages, crinkly wrapping paper, finger tapping), watching someone perform repetitive mundane actions (e.g. folding towels, going through items in a handbag), watching someone closely inspecting day-to day objects, hearing whisper, watching someone’s hair being brushed or watching videos with various role plays (visit a doctor, spa or a shop). ASMR is not frisson (e.g. ‘goosebumps’ when listening to music).

| **1** | Having read the description would you say you ever experience ASMR whether it is from watching such videos or in everyday life? Would you classify yourself as somebody who experiences ASMR? | **Yes** | **No**  **Skip to the Absorption Questionnaire** |
| --- | --- | --- | --- |
| **2** | Do you watch ASMR videos? | **Yes** | **No**  **Skip to 6** |
| **3** | Why do you watch ASMR videos? Is it to experience the tingles, relax, fall asleep. Please Specify | **____** | |
| **4** | How many ASMR videos do you typically watch in a single session? | **____** | |
| **5** | How often do you watch ASMR videos? | Drop down box (never, less than once a month, 2-3 times a month, 2-3 times a week, daily) | |
| **6** | Do you require specific conditions to achieve ASMR? | **Yes** | **No**  **Skip to 8** |
| **7** | Please briefly describe the conditions you require to achieve ASMR sensations (e.g. busy, quiet room, bright lighting etc). | **___** | |
| **8** | Please indicate whether or not these common stimuli trigger your ASMR – please select all the items that trigger your tingling sensations while viewing ASMR videos or in your daily life.  **DUE TO A TECHNICAL ISSUE WITH THE SURVEY WHICH DIDN'T ALLOW TO TICK MORE THAN ONE ANSWER THIS QUESTION HAD TO BE DISCARDED FROM ANALYSIS** | Multiple choice: Crisp sounds (tapping/crinkling), personal attention, whispering, role-plays (e.g. visit to a hairdresser, spa, doctor), hair brushing, paying attention to detail, slowly performing mundane actions or explaining something, or concentrating on something, people eating, other (please elaborate) | |

**9.** Please indicate how intense the ASMR experience is on average when engaging with those stimuli.

Intensity Scale (higher numbers represent increasing intensity): 0 = No Tingles 1 2 3 = Moderately Intense 4 5 6 = Most Intense ASMR Experience

For example, if whispering triggers the most intense tingles for someone, they would label that stimulus a "6".

| Stimulus | Intensity (please tick the appropriate number according to the scale above) |
| --- | --- |
| crisp sounds (tapping/crinkling) | 0 1 2 3 4 5 6 |
| personal attention | 0 1 2 3 4 5 6 |
| whispering | 0 1 2 3 4 5 6 |
| role-plays (e.g. visit to a hairdresser, spa, doctor) | 0 1 2 3 4 5 6 |
| hair brushing | 0 1 2 3 4 5 6 |
| paying attention to detail, concentrating on something, slowly performing mundane actions or explaining something | 0 1 2 3 4 5 6 |
| people eating | 0 1 2 3 4 5 6 |
| other (please elaborate) | 0 1 2 3 4 5 6 |

**10**. Does the intensity of these tingles vary from session to session of ASMR video viewing? (Yes/No)

**11.** On average, how pleasurable is an ASMR experience?

a) Quite Uncomfortable

b) Mildly Uncomfortable

c) Neutral

d) Mildly pleasurable

e) Quite pleasurable

**Tellegen Absorption Scale**

Please select the degree to which you agree with the following statements on a sale of 1 (Disagree Strongly) – 5 (Agree Strongly)

1. Sometimes I feel and experience things as I did when I was child.
2. I can be greatly moved by eloquent or poetic language.
3. While watching a movie, TV show or a play, I may become so involved that I may forget about myself and my surrounding and experience the story as if it were real and as if I were taking part in it.
4. If I stare at a picture and then look away from it, I can sometime “see” an image of the picture almost as I were still looking at it.
5. Sometimes I feel as if my mind could envelop the whole world.
6. I like to watch cloud shapes change in the sky.
7. If I wish, I can imagine (or daydream) some things so vividly that they hold my attention as a good movie or story does.
8. I think I really know what some people mean when they talk about mystical experiences.
9. I sometimes “step outside” my usual self and experience an entirely different state of being.
10. Textures – such as wool, sand, wood – sometimes remind me of colours or music.
11. Sometimes I experience thing as if they were doubly real.
12. When I listen to music I can get so caught up in it that I don’t notice anything else.
13. If I wish, I can imagine that my body is so heavy that I could not move it.
14. I can often somehow sense the presence of another person before I actually see or hear them.
15. The crackle and flames of a wood fire stimulate my imagination.
16. It is sometimes possible for me to be completely immersed in nature or in art and to feel as if my whole state of consciousness has somehow been temporarily altered.
17. Different colours have distinctive and special meanings for me.
18. I am able to wander off into thoughts while doing a routine task and actually forget that I am doing the task, and then find a few minutes later than I have completed it.
19. I can sometimes recollect certain past experiences in my life with such clarity and vividness that is like living them again or almost so.
20. Things that might seem meaningless to others often make sense to me.
21. While acting a play I think I could really feel the emotions of the character and “become” her/him for the time being, forgetting both myself and the audience.
22. My thoughts often don’t occur as words but as visual images.
23. I often take delight in small things (such as the colours in soap bubbles).
24. When listening to organ music or other powerful music I sometimes feel as if I am being lifted into the air.
25. Sometimes I can change noise into music by the way I listen to it.
26. Some of my most vivid memories are called up by scents and smells.
27. Some music reminds me of pictures or changing colour patterns.
28. I often know what someone is going to say before he or she says it.
29. I often have “physical memories”, for example, after I have been swimming I may still feel as if I am in the water.
30. The sound of a voice can be so fascinating to me that I can just go on listening to it.
31. At times I somehow feel the presence of someone who is not physically there.
32. Sometimes thoughts and images come to me without the slightest effort on my part.
33. I find that different odours have different colours.
34. I can be deeply moved by a sunset.

**Mindful Awareness and Attention Questionnaire**

Below is a collection of statements about your everyday experience. Using the 1 (Almost Always) – 6 (Almost Never) scale below, please indicate how frequently or infrequently you currently have each experience. Please answer according to what really reflects your experience rather than what you think your experience should be. Please treat each item separately from every other item.

1. I could be experiencing some emotion and not be conscious of it until sometime later.

2. I break or spill things because of carelessness, not paying attention, or thinking of something else.

3. I find it difficult to stay focused on what’s happening in the present.

4. I tend to walk quickly to get where I’m going without paying attention to what I experience along the way.

5. I tend not to notice feelings of physical tension or discomfort until they really grab my attention.

6. I forget a person’s name almost as soon as I’ve been told it for the first time.

7. It seems I am “running on automatic,” without much awareness of what I’m doing.

8. I rush through activities without being really attentive to them.

9. I get so focused on the goal I want to achieve that I lose touch with what I’m doing right now to get there.

10. I do jobs or tasks automatically, without being aware of what I'm doing.

11. I find myself listening to someone with one ear, doing something else at the same time. 12. I drive places on ‘automatic pilot’ and then wonder why I went there.

13. I find myself preoccupied with the future or the past.

14. I find myself doing things without paying attention.

15. I snack without being aware that I’m eating.

**Flow Experiences Questionnaire**

Please read the following quotes:

My mind isn’t wandering. I am not thinking of something else. I am totally involved in what I am doing. My body feels good. I don’t seem to hear anything. The world seems to be cut off from me. I am less aware of myself and my problems. My concentration is like breathing I never think of it. When I start, I really do shut out the world. I am really quite oblivious to my surroundings after I really get going. I think that the phone could ring, and the doorbell could ring or the house burn down or something like that. When I start I really do shut out the world. Once I stop I can let it back in again. I am so involved in what I am doing. I don’t see myself as separate from what I am doing.

Have you ever felt similar experiences? Yes/No

If yes, what activities were you engaged in when you had such experiences? Please write the name of the activity – among those you quoted if any – which best represents the experience described in the three quotations, that is, the activity where you feel this experience with the highest intensity.

___

How often do you experience it? ___

Please rate how you feel when you engage in this activity on a 7-point scale ranging from 1 (not at all characteristic of me) – 7 (very characteristic of me).

When I engage in this activity:

1. I feel I have clear goals
2. I feel self-conscious
3. I feel in control
4. I lose track of time
5. I feel I know how well I am doing
6. I have a high level of concentration
7. I forget personal problems
8. I feel fully involved
